# Low incidence of tumor lysis syndrome in elderly patients with chronic lymphocytic leukemia treated with venetoclax under real-world conditions: results from the prospective observational VeRVe study

**DOI:** 10.1007/s00277-024-05638-7

**Published:** 2024-02-29

**Authors:** Ingo Schwaner, Thomas Kuhn, Christoph Losem, Thomas Wolff, Burkhard Otremba, Matthias Zaiss, Johannes Hülsenbeck, Kirsten Famulla, Thomas Nösslinger, Davide Rossi

**Affiliations:** 1 Onkologische Schwerpunktpraxis Kurfuerstendamm, Kurfuerstendamm 65, 10707 Berlin, Germany; 2 AbbVie Deutschland GmbH & Co. KG, Hämatologie, Wiesbaden, Germany; 3 Rheinlandklinikum Grevenbroich, Grevenbroich, Germany; 4 Onkologie Lerchenfeld, Hamburg, Germany; 5 Onkologische Praxis Oldenburg, Oldenburg, Germany; 6 Praxis für interdisziplinäre Onkologie & Hämatologie, Freiburg, Germany; 7 3rd Medical Department for Hematology and Oncology, Hanusch Krankenhaus, Wien, Austria; 8 Clinic of Hematology, Oncology Institute of Southern Switzerland (IOSI), Bellinzona, Switzerland; 9 Experimental Hematology, Institute of Oncology Research, Bellinzona, Switzerland; 10 Università della Svizzera italiana, Lugano, Switzerland

**Keywords:** Venetoclax, Chronic lymphocytic leukemia, Tumor lysis syndrome, Real-world evidence

## Abstract

Venetoclax is active in both frontline and relapsed/refractory settings for the treatment of chronic lymphocytic leukemia (CLL). Although the prevalence and severity of tumor lysis syndrome (TLS) are well characterized in clinical trials, laboratory and clinical TLS remain relatively unexplored in real-world clinical practice.

In this prospective, real-world observational study, we aimed to determine the incidence and outcomes of TLS in patients with CLL receiving venetoclax outside a clinical trial. The study (VeRVe) was conducted in centers in Austria, Germany, and Switzerland.

Two hundred and thirty-nine patients were treated according to local label with at least one dose of venetoclax. Patient demographics, baseline characteristics, and blood chemistry at baseline were documented, and descriptive statistical analyses were conducted.

Seventy eight patients (33%) were treated with venetoclax monotherapy, 101 (42%) with venetoclax in combination with rituximab and 60 (25%) with venetoclax in combination with obinutuzumab. In all cases, the TLS risk mitigation strategy adhered to the ramp-up protocol. Median age was 73 years and 66% of patients were male. The majority of patients (75%) had relapsed/refractory CLL, 63/192 (32.8%) patients tested had a del(17p) and 93/134 (69.4%) patients tested had unmutated immunoglobulin heavy chain variable region gene (IGHV). Clinical TLS occurred in 5 patients (2.1%) and laboratory TLS occurred in 15 patients (6.3%). Ten patients received specific treatment, of which 6 were hospitalized. There were no deaths due to a TLS event and venetoclax was well-tolerated. Of the 5 clinical TLS events reported, none were fatal or resulted in renal failure (NCT03342144, registered on Nov 10, 2017).

**Supplementary Information:**

The online version contains supplementary material available at 10.1007/s00277-024-05638-7.

## Introduction

Venetoclax is a first-in-class, potent and highly selective, orally bioavailable, B-cell lymphoma-2 homology 3 (BH3)-mimetic antagonist of B-cell lymphoma 2 (BCL2), an anti-apoptotic protein constitutively overexpressed in chronic lymphocytic leukemia (CLL). BCL2 is responsible for the resistance to *TP53-*mediated apoptosis by sequestering a surplus of pro-apoptotic BH3-only proteins, favoring cell survival. Venetoclax induces apoptosis by BCL2 inhibition [1, 2]. It binds to the BH3 domain of BCL2 with subsequent release of sequestered BH-3-only proteins, thereby triggering BAX/BAK-mediated rapid tumor-cell death and anti-tumor activity [3–8].

Venetoclax is approved for several clinical situations in CLL. First, it is approved as monotherapy for the treatment of patients with CLL with a 17p deletion or *TP53* mutation who cannot be treated with a B-cell receptor inhibitor or if these medicines have stopped working, and for patients without these genetic alterations who have failed chemoimmunotherapy and a B‑cell receptor pathway inhibitor [9]. Second, venetoclax is approved in combination with the anti-CD20 antibody rituximab (VenR), as a fixed-duration regimen for pre-treated patients with CLL [10]. Third, venetoclax is approved in combination with obinutuzumab (VenO) or ibrutinib (VenI) in previously untreated patients with CLL [11–13].

Due to its potent apoptotic effect on lymphocytic cells, venetoclax treatment is associated with a risk for tumor lysis syndrome (TLS). TLS is a potentially serious oncologic emergency that can be triggered by spontaneous or therapy-induced rapid cell death [14, 15] The rapid release of cell contents can lead to hyperkalemia, hypocalcemia, hyperphosphatemia, and hyperuricemia, which may result in renal dysfunction, cardiac arrhythmias, and encephalopathy. In extreme cases, TLS can lead to seizures and death from multiorgan failure [16, 17]. TLS can be classified according to the Howard or Cairo-Bishop criteria as either laboratory or clinical TLS [18, 19]. Laboratory TLS according to the Howard criteria is present when two or more metabolic disturbances (hyperuricemia, hyperkalemia, hyperphosphatemia, or hypocalcemia) occur within a 24-hour period. Clinical TLS is present when, in addition to the laboratory TLS changes, other changes such as elevated creatinine, seizures, cardiac arrhythmias, or death occur [18].

In the first-in-human/phase 1b trials of venetoclax, TLS was reported as a serious side effect. In order to minimize the risk of TLS, a strict TLS risk stratification was developed along with dose ramp-up (20 mg, 50 mg, 100 mg, 200 mg,

400 mg) over 5 weeks (dose titration phase) [20]. Adherence to this dosing strategy, combined with TLS prophylaxis with hypouricemic drugs, hydration, and monitoring of TLS markers has allowed venetoclax to be safely administered in clinical trials [9–12].

A comprehensive review of TLS incidence across venetoclax clinical trials demonstrated that patients with a high tumor burden (bulky lymph nodes ≥ 5 cm and/or elevated absolute lymphocyte count [ALC]) are at a higher risk when initiating venetoclax. Reduced renal function at screening (creatinine clearance < 80 mL/min) and concomitant medications such as CYP3A4 inhibitors further predispose patients to the risk of developing TLS [17, 21–23].

By following the recommended measures after initiating venetoclax, the incidence of laboratory-confirmed TLS within clinical trials in CLL was ∼1.1–3.8% with no cases of clinical manifestation [22, 24]. In contrast, retrospective real-world observations have reported higher rates of laboratory (5.7–6.3%) and clinical (2.7–6.3%) TLS events [25–27].

To determine the effectiveness and tolerability of venetoclax as monotherapy (Ven), or in combination with rituximab (VenR) or obinutuzumab (VenO) in patients with CLL under real-world conditions, we conducted a prospective non-interventional observational study (VeRVe) within a real-world clinical setting. These post-hoc analyses were conducted to determine the incidence and outcome of TLS events in these patients. The patient case reports discussed in the current analyses were documented during the study and clinical TLS events were reviewed and discussed in the context of patient-specific disease characteristics, venetoclax treatment initiation, and TLS risk-mitigation.

## Methods

### Study design and population

VeRVe is a prospective, international, multi-center observational study in patients with CLL who receive venetoclax as single agent or in combination with an anti-CD20 antibody. The study is being conducted across Austria, Germany, and Switzerland, and was designed to record and analyze real-world treatment data from daily clinical practice. All processes, treatments, procedures (i.e., blood sampling), and diagnostic steps, including the times for follow-up examinations, were selected to correlate with daily routine practice. Informed consent was obtained from all participants included in the study.

### Procedures

Adult patients with CLL requiring therapy according to International Workshop on Chronic Lymphocytic Leukemia criteria and who initiated venetoclax therapy were eligible if treated as specified in the local label for any line of treatment. The regimen was prescribed at the discretion of the treating physician in accordance with local clinical practice. Treatment decisions were made independently and preceded the decision to offer the patient the opportunity to participate in this study.

All patient and disease characteristics were summarized descriptively at baseline prior to initiation of venetoclax therapy. Quantitative variables are presented as absolute number, mean, median, standard deviation, quartiles, minimum and maximum. Categorical/binary variables, such as sex, age groups, initial stage, etc., are presented by absolute numbers and percentages. TLS events were documented by the physician and recorded as laboratory and/or clinical TLS according to the definition by Howard et al. [18]. The study was conducted in accordance with local laws and regulations of the participating countries, and all patients signed informed consent.

### Statistical analyses

The study is ongoing at the time of this publication and the current analyses are based on a data cut-off from March 3, 2022. In these descriptive, retrospective analyses, adult patients with CLL requiring therapy who have received at least one dose of Ven, VenR, or VenO were included. Pearson’s method was used to test for a correlation between the occurrence of isolated metabolic abnormalities and TLS. Statistical analyses were performed using SAS version 9.4 (SAS Institute Inc., Cary, NC, USA) and R version 4.1.3 (R Foundation for Statistical Computing, Vienna, Austria) by the German Oncology group.

## Results

### Patient characteristics

A total of 239 patients who had received at least one dose of venetoclax were included in the current analyses. Seventy-eight (33%) patients were treated with Ven, 101 (42%) patients were treated with VenR and 60 (25%) received VenO. Median age at baseline was 74 and 73 years for the Ven and VenR groups, respectively, and 67 years in the VenO group. Patients treated with Ven or VenR received a median of 2 and 1 prior lines of treatment, respectively (range, 1–10). Prognostically unfavorable cytogenetics (i.e., del(17p)/*TP53*^mut^) were more common in the group treated with Ven vs. VenR or VenO (del[17p]: 44% vs. 28% or 24%; *TP53*^mut^: 49% vs. 30% or 15%, respectively) amongst those tested. Of those tested, the proportion of patients with unmutated IGHV was 73%, 69%, and 67% in the Ven, VenR, and VenO groups, respectively. Baseline characteristics prior to venetoclax treatment initiation are shown in Table 1.

**Table 1:** Baseline Characteristics

	Total (*N* = 239)	Ven (*N* = 78)	VenR (*N* = 101)	VenO (*N* = 60)
Sex, n				
Male	157	47	73	37
Female	82	31	28	23
Age, years, median [range]	73 [40–94]	74 [40–94]	73 [50–88]	67 [43–87]
Binet stage^#^, n (%)				
A	54 (23)	21 (27)	21 (21)	12 (20)
B	92 (39)	28 (36)	37 (37)	27 (45)
C	92 (39)	29 (37)	43 (43)	20 (33)
Missing	1	0	0	1
Del(17p), n (%)*				
Deleted	63 (26)	30 (38)	23 (23)	10 (17)
Not deleted	129 (54)	38 (49)	59 (58)	32 (53)
Missing	47 (20)	10 (13)	19 (19)	18 (30)
*TP53*, n (%)*				
Mutated	62 (26)	31 (40)	24 (24)	7 (12)
Unmutated	127 (53)	32 (41)	56(55)	39 (65)
Missing	50 (21)	15 (19)	21 (21)	14 (23)
IGHV status, n (%)*				
Mutated	41 (17)	10 (13)	18 (18)	13 (22)
Unmutated	93 (39)	27 (35)	40 (40)	26 (43)
Missing	105 (44)	41 (53)	43 (43)	21 (35)
Prior therapies, n	179	78	101	0
Median [range]	2 [1–10]	2 [1–10]	1 [1–10]	0
Patients with comorbidities, n (%)	177 (74)	62 (26)	77 (32)	38 (16)

*Percentage of patients tested

^#^Binet staging system for CLL: Stage A, fewer than 3 groups of swollen (enlarged) lymph nodes. Stage B, 3 or more groups of enlarged lymph nodes. Stage C, low number of red blood cells or platelets

Del: deletion; IGHV: immunoglobulin heavy chain variable region gene; Ven: venetoclax monotherapy; VenR: venetoclax and rituximab; VenO: venetoclax and obinutuzumab

Baseline characteristics of patients with (*n* = 28) or without (*n* = 200) occurrence of TLS are shown in Table 2. Generally, comorbidities were similar between both groups. Median leukocyte and absolute lymphocyte counts were higher in the TLS group compared with the non-TLS group (63 vs. 19.3 × 10^9^/L and 30.9 vs. 10.9 × 10^9^/L, respectively). Higher rates for both parameters were also observed among the 5 patients that developed clinical TLS. The median creatinine clearance was higher in the non-TLS group (Pearson’s method, *p* = 0.03). Data for lymph node size was not available for all patients, but the percentage of patients with lymph node size > 5 cm was comparable in patients with or without TLS (17.9% vs. 18.5%, respectively). The percentage of patients who received allopurinol as a supportive agent at baseline was higher in the non-TLS compared with the TLS group (61.5% vs. 53.6%). Similarly, slightly more patients who experienced clinical TLS had received allopurinol at baseline compared with those patients with laboratory TLS (60.0% vs. 54.2%, respectively).

**Table 2:** Summary of baseline parameters between TLS and non-TLS patients

Parameter	All patients	Patients without TLS*	Patients with TLS*	Patients with laboratory TLS	Patients with clinical TLS
Patients, n/N	239	200	28	24	5
Age, years, median [range]	73 [40–94]	73 [40–94]	75 [54–88]	74.5 [54–84]	76 [54–88]
Leukocytes, ×10^9^/L, median [range]	*N* = 229 19.3 [1.4–407]	*N* = 194 19.3 [1.4–407]	*N* = 26 63 [6.4–254.8]	*N* = 22 46 [6.4–254.8]	*N* = 5 121 [44–242]
Lymphocytes, ×10^9^/L, median [range]	*N* = 175 10.9 [0.2–287.1]	*N* = 149 10.9 [0.2–287.1]	*N* = 19 30.9 [0.8–125]	*N* = 16 30.2 [0.8–125]	*N* = 3 69.9 [0.8–94]
Creatinine, mg/dL, median [range]	*N* = 229 1.02 [0.42–3.06]	*N* = 194 1.01 [0.42–3.06]	*N* = 26 1.2 [0.7–2.3]	*N* = 22 1.3 [0.7–2.3]	*N* = 5 1.2 [1.0–1.4]
Creatinine clearance, mL/min, median, [range]	*N* = 225 68.2 [20.3–214.6]	*N* = 192 69.3 [20.3–214.6]	*N* = 26 56.7 [25.9–104.4]	*N* = 22 59.4 [25.9–104.4]	*N* = 5 59 [33.2–76.9]
Potassium, mmol/L, median [range]	*N* = 222 4.3 [2.26–5.9]	*N* = 188 4.3 [2.26–5.7]	*N* = 25 4.4 [3.4–5.9]	*N* = 21 4.4 [3.4–5.9]	*N* = 5 4.3 [3.8–4.9]
Calcium, mmol/L, median [range]	*N* = 210 2.3 [0.53–4.8]	*N* = 177 2.3 [0.53–4.8]	*N* = 25 2.3 [2.01–2.6]	*N* = 21 2.3 [2.02–2.6]	*N* = 5 2.3 [2.0–2.5]
Phosphate, mmol/L, median [range]	*N* = 137 1.08 [0.5–1.95]	*N* = 114 1.08 [0.58–1.95]	*N* = 18 1.1 [0.5–1.4]	*N* = 15 1.1 [0.5–1.4]	*N* = 4 1.2 [0.8–1.4]
Del(17p)/*TP53*^mut^, n/N (%)	85/215 (39.5)	72/179 (40)	11/27 (41)	11/23 (47.8)	0/5 (0)
IGHV^unmut^, n/N (%)	93/134 (69.4)	79/200 (39.5)	12/12 (100)	11/11 (100)	1/1 (100)
Lymph node size					
Patients assessed, n (%)	94 (39.3)	82 (41.0)	10 (35.7)	8 (33.3)	2 (40.0)
Size > 5 cm, n (%)	42 (17.6)	37 (18.5)	5 (17.9)	5 (20.8)	0
Comorbidities, n					
Any	182	148	22	19	4
Renal	28	21	5	4	1
Cardiovascular	128	108	15	15	0
Metabolic/Endocrine	74	60	10	9	1
Supportive medication, n					
Any	172	129	15	14	1
TLS prophylaxis, n					
Allopurinol	162	123	15	13	3
Rasburicase	10	8	2	1	0
Febuxostat	5	4	1	1	1

*Patients who had documented laboratory parameters at baseline, no data available for 11 patients

Del: deletion; IGHV: immunoglobulin heavy chain variable region gene; TLS: tumor lysis syndrome

### Incidence of TLS

Twenty-eight (15.6%) cases of TLS were identified. Of these, 19 (10.6%) were documented as TLS in the electronic case report form (eCRF) and 9 (5%) were not documented as TLS but fulfilled the Howard criteria for TLS (Table 3). The incidence of laboratory TLS was higher in the VenR cohort compared with the Ven cohort (10.9% vs. 5%), whereas the rate of clinical TLS was lower in the VenR cohort compared with the Ven cohort (2% vs. 3.8%). As no cases of TLS were observed in the VenO arm and Obinutuzumab is dosed before Ven, resulting in debulking this subgroup of patients were excluded from the calculation of the incidence of TLS.

**Table 3:** Incidence of TLS

Event	Total	Ven	VenR
TLS events, n (%)	28 (11.7)^#^	12 (15.3)	16 (15.8)
Laboratory TLS*^,#^	24 (10)^#^	9 (11.5)	15 (14.9)
Clinical TLS	5 (2.1)	3 (3.8)	2 (2)
With treatment	10 (52.6)	4 (57)	6 (50)
With hospitalization	6 (31.6)	1 (14.3)	5 (41.7)
Event	Total	Ven	VenR
TLS events, n (%)	28 (15.6)^#^	12 (15.3)	16 (15.8)
Laboratory TLS*^,#^	15 (8.4)^#^	4 (5)	11 (10.9)
Clinical TLS	5 (2.8)	3 (3.8)	2 (2)
With treatment	10 (52.6)	4 (57)	6 (50)
With hospitalization	6 (31.6)	1 (14.3)	5 (41.7)

*Cases with blood chemistry values that fulfilled the Howard criteria

^#^Including one patient with two laboratory TLS events

TLS: tumor lysis syndrome; Ven: venetoclax monotherapy; VenR: venetoclax and rituximab; VenO: venetoclax and obinutuzumab

#### Laboratory TLS

Overall, the test results of 15 patients (8.4% of the eligible population) fulfilled the Howard criteria for laboratory TLS alone. One patient developed two laboratory TLS events; the first occurring 2 days after the first dose of 20 mg, and the second 32–33 h after the first 200 mg dose.

#### Clinical TLS

Of the 19 TLS events which were documented in the eCRF, 5 met the criteria for clinical TLS. Three of these events occurred in patients treated with Ven and 2 cases in patients treated with VenR. Three patients required hospitalization during treatment (Ven:1; VenR:2). The median hospitalization time was 17 days. One patient already had an elevated uric acid level at therapy initiation. For the other 4 patients, blood chemistry was without remarkable findings at baseline. Table 3 gives an overview of the patient-specific characteristics for patients who experienced a clinical TLS.

**Table 4:** Patients with clinical TLS

Patient ID	Dose at which TLS occurred, mg	Regimen	Hospitalized	Lymphocytes, cells/µL*	Leukocytes, cells/µL*	Creatinine clearance, mL/min*	Age, years	Short ramp-up	Discontinued
1	100	Ven	No	94,000	242.0	34.4	80	Yes	No
2	50	VenR	Yes, day 24	N/A	120.6	65.8	54	Yes	No
3	20	Ven	Yes, day 17	840	44.3	60.8	76	No	Yes
4	20	VenR	Yes, day 8	N/A	139.0	45.3	88	No	Yes
5	20	Ven	No	69,900	78.9	79.2	71	No	No

*At baseline. Normal range for lymphocytes is 1,500–3,000 cells/µL and leukocytes is 4,000–10,000 cells/µL. For creatinine, a value between 1–1.5 mL/min corresponds to normal renal function

TLS: tumor lysis syndrome; Ven: venetoclax monotherapy; VenR: venetoclax and rituximab

TLS occurred in all patients during the early ramp-up phase, none of which was fatal or led to renal failure. No TLS event was documented as the reason for discontinuation of venetoclax. However, two patients with clinical TLS discontinued venetoclax treatment early due to other AEs (1 thrombocytopenia and 1 arrythmia). Three patients reached the final dose of 400 mg venetoclax, despite clinical TLS during the early ramp-up phase. For one patient with clinical TLS who reached the final dose of 400 mg, the ramp-up phase was shortened before and during occurrence of the TLS.

## Discussion

In the current analyses, 239 patients received at least one dose of venetoclax. Under real-world conditions, venetoclax given as monotherapy or in combination with rituximab is mainly prescribed to elderly pretreated patients. The median age of the patient population in the monotherapy and VenR combination arms was higher at baseline than in the pivotal monotherapy (M13-982, M14-032) [9, 28] and combination therapy trials (MURANO) [10] but younger in the VenO combination arm than the pivotal frontline therapy trial (CLL14) [29]. However, the median age at diagnosis (62 years) in this study was lower than the literature data published by Surveillance, Epidemiology, and End Results (SEER) Program (70 years) [30]. This indicates a longer duration of disease in our cohort. Adverse risk cytogenetics (del[17p]/*TP53*^mut^) were more common in the monotherapy group than in the combination groups, although they were generally comparable to previous real-world studies [26, 31].

The overall incidence of TLS in this study was 15.6%. In a large retrospective analysis, Seymour et al. [32] examined the rate of TLS cases in patients with CLL who received venetoclax-based regimens in frontline and relapsed/refractory diseases. Of 1,138 patients from eight clinical trials, 20 TLS (1.8%) events of any grade and 50 blood chemistry abnormalities, which met the Howard criteria of TLS, were reported. All TLS cases were transient, allowing affected patients to resume/continue therapy without permanent sequelae or death due to TLS. In contrast to data from clinical trials, the incidence rate of documented TLS cases (of any grade) within the real-world studies across the US and UK ranged from 4 to 15% [25, 27, 33]. Clinical TLS rates ranged from 2 to 6% [27, 33]. Across Europe, TLS was observed in 14 (22%) patients (clinical: 2 [3%]) receiving Ven in a French Innovative Leukemia Organization (FILO) study of the French compassionate use cohort [31]; in a Spanish retrospective observational trial (VENARES), 125 eligible patients treated with Ven (*n* = 71), VenR (*n* = 36), VenO (*n* = 5) or other Ven combination.

(*n* = 13) were included in the analysis. TLS was reported in 4 (3.2%) patients during ramp up (3 laboratory, 1 clinical) with no patients discontinuing treatment due to TLS [34]. Thus, the overall documented TLS rate of 15.6% and clinical TLS rate of 2.8% (5/179 patients) found in this analysis is comparable to other retrospective real-world studies and prospective clinical trial data. In the study by Seymour et al. [32], authors analyzed additional data from the AbbVie Global Pharmacovigilance Database including post-marketing data from approximately 20,000 patients treated under real-world conditions. A total of 236 cases of TLS were reported including 17 cases with severe consequences (clinical sequelae) attributable to TLS. These patients had relapsed/refractory CLL and a median age of 69 years (range, 45–84). Multiple comorbidities were present, including renal insufficiency (*n* = 7), defined as creatinine clearance or glomerular filtration rate < 80 mL/min (at baseline) and/or reported history of renal impairment. Of the 17 severe cases (~ 0.1%), 13 were fatal and 4 required dialysis. Eight patients developed TLS at the first dose step of 20 mg. The TLS risk category at baseline was not reported for 7 patients. Despite having enough data on the 17 cases to conclude that the severe or fatal outcomes were attributable to TLS, some details regarding these cases were unavailable. The authors concluded that key factors contributing to TLS may have included non-adherence to label recommendations for TLS risk mitigation by the prescribers [32]. It is therefore important that prescribers follow recommended preventive measures, including risk assessment, guidelines on hydration and use of anti-hyperuricemic agents, blood chemistry monitoring with early intervention when needed, and dose modifications for drug-drug interactions.

In our study, no fatal TLS events were reported, and all cases were transient. Most patients resumed therapy without any irreversible sequelae. The majority of the clinical TLS events occurred at the early phase of dose escalation (20/50 mg). All patients had impaired renal function (GFR < 80 mL/min) at baseline which was also significantly lower in patients with compared to patients without a TLS event. Lymphocytes were increased at baseline, which is considered the most relevant predisposing factor for TLS. Although ALC and lymph node size are the only parameters currently deemed necessary to predict risk of TLS per label, previous analyses have demonstrated that baseline creatinine clearance can serve as a predictor of TLS [25]. In our study, no other baseline parameters were found to be predictive of the incidence of TLS. Additional measures of tumor bulk may also be important in assessing risk for TLS, including spleen size, extent of bone marrow involvement by CLL, serum lactate dehydrogenase, and β-2 microglobulin [27].

Based on available data and analyses, particularly from the real-world/post-marketing retrospective studies, the venetoclax summary of product characteristics (SmPC) was revised in June 2021 to reflect the updated recommendations and to emphasize the importance of strict adherence to the TLS mitigation measures in all CLL patients [13]. To minimize the risk, prescribing physicians should assess patient-specific factors for TLS risk, including comorbidities and particularly impaired renal function, tumor burden, and splenomegaly. Prophylactic fluid intake and the use of uric acid lowering medication (e.g., allopurinol) is mandatory in all patients prior to the first dose of venetoclax. Laboratory blood chemistry values and tumor burden should be closely monitored, and the recommended dose adjustments and actions should be followed in the event of changes in blood counts or symptoms suggestive of TLS. These data allow for a richer understanding of the rates of clinical and laboratory TLS with venetoclax outside of the clinical trial setting.

In summary, multiple factors may give rise to the risk of TLS. Under real-world conditions in Austria, Germany, and Switzerland, venetoclax therapy initiation was well-tolerated and few clinical TLS events occurred, and none of them were fatal or lead to total renal failure. These findings highlight the importance of continuing to follow the current TLS mitigation protocol, i.e., the five-week ramp-up, patient hydration, anti-hyperuricemic prophylaxis, and close monitoring of the patients’ blood chemistry in order to prevent the occurrence of a TLS event. Further research into biochemical factors that might be associated with TLS risk is warranted.

Future observations from this ongoing study will add to the body of evidence for use of TLS mitigation measures with venetoclax treatment as mono- and combination-therapy.

### Electronic supplementary material

Below is the link to the electronic supplementary material.

Supplementary Material 1
