# Supplementary material for: Low incidence of tumor lysis syndrome in elderly patients with chronic lymphocytic leukemia treated with venetoclax under real-world conditions: results from the prospective observational VeRVe study
Source: Ann Hematol. 2024 Feb 29;103(6):2013–20. doi: 10.1007/s00277-024-05638-7 (PMC11090950; doi:10.1007/s00277-024-05638-7)
Supplement: Supplementary file 1 — Supplementary Material 1 [file 277_2024_5638_MOESM1_ESM.docx]

**Low incidence of tumor lysis syndrome in elderly patients with chronic lymphocytic leukemia treated with venetoclax under real-world conditions: Results from the prospective observational VeRVe study**

Ingo Schwaner^a^, Thomas Kuhn^b^, Christoph Losem^c^, Thomas Wolff^d^, Burkhard Otremba^e^, Matthias Zaiss^f^, Johannes Hülsenbeck^b^, Kirsten Famulla^b^, Thomas Nösslinger^g^, Davide Rossi^h,i,j^

^a^ Onkologische Schwerpunktpraxis Kurfuerstendamm, Kurfuerstendamm 65, 10707
Berlin, Germany

^b^ AbbVie Deutschland GmbH & Co. KG, Hämatologie, Wiesbaden, Germany

^c^ Rheinlandklinikum Grevenbroich, Grevenbroich, Germany

^d^ Onkologie Lerchenfeld, Hamburg, Germany

^e^ Onkologische Praxis Oldenburg, Oldenburg, Germany

^f^ Praxis für interdisziplinäre Onkologie & Hämatologie, Freiburg, Germany

^g^ 3rd Medical Department for Hematology and Oncology, Hanusch Krankenhaus, Wien, Austria
^h^ Clinic of Hematology, Oncology Institute of Southern Switzerland (IOSI),
Bellinzona, Switzerland

^i^ Experimental Hematology, Institute of Oncology Research, Bellinzona, Switzerland
^j^ Università della Svizzera italiana, Lugano, Switzerland

* Corresponding author: Schwaner I., D-10707 Berlin, Germany. Ph.: +49-30 887742570

E-mail address: ingo.schwaner@onkologie-kurfuerstendamm.de

**Journal: Annals of Hematology**

# Abstract

Venetoclax is active in both frontline and relapsed/refractory settings for the treatment of chronic lymphocytic leukemia (CLL). Although the prevalence and severity of tumor lysis syndrome (TLS) are well characterized in clinical trials, laboratory and clinical TLS remain relatively unexplored in real-world clinical practice.

In this prospective, real-world observational study, we aimed to determine the incidence and outcomes of TLS in patients with CLL receiving venetoclax outside a clinical trial. The study (VeRVe) was conducted in centers in Austria, Germany, and Switzerland.

Two hundred and thirty-nine patients were treated according to local label with at least one dose of venetoclax. Patient demographics, baseline characteristics, and blood chemistry at baseline were documented, and descriptive statistical analyses were conducted.

Seventy eight patients (33%) were treated with venetoclax monotherapy, 101 (42%) with venetoclax in combination with rituximab and 60 (25%) with venetoclax in combination with obinutuzumab. In all cases, the TLS risk mitigation strategy adhered to the ramp-up protocol. Median age was 73 years and 66% of patients were male. The majority of patients (75%) had relapsed/refractory CLL, 63/192 (32.8%) patients tested had a del(17p) and 93/134 (69.4%) patients tested had unmutated immunoglobulin heavy chain variable region gene (IGHV). Clinical TLS occurred in 5 patients (2.1%) and laboratory TLS occurred in 15 patients (6.3%). Ten patients received specific treatment, of which 6 were hospitalized. There were no deaths due to a TLS event and venetoclax was well-tolerated. Of the 5 clinical TLS events reported, none were fatal or resulted in renal failure (NCT03342144, registered on Nov 10, 2017).

*Keywords:* Venetoclax*,* chronic lymphocytic leukemia, tumor lysis syndrome, real-world evidence

# Introduction

Venetoclax is a first-in-class, potent and highly selective, orally bioavailable, B-cell lymphoma-2 homology 3 (BH3)-mimetic antagonist of B-cell lymphoma 2 (BCL2), an anti-apoptotic protein constitutively overexpressed in chronic lymphocytic leukemia (CLL). BCL2 is responsible for the resistance to *TP53-*mediated apoptosis by sequestering a surplus of pro-apoptotic BH3-only proteins, favoring cell survival. Venetoclax induces apoptosis by BCL2 inhibition [1, 2]. It binds to the BH3 domain of BCL2 with subsequent release of sequestered BH-3-only proteins, thereby triggering BAX/BAK-mediated rapid tumor-cell death and anti-tumor activity [3, 4, 5, 6, 7, 8].

Venetoclax is approved for several clinical situations in CLL. First, it is approved as monotherapy for the treatment of patients with CLL with a 17p deletion or *TP53* mutation who cannot be treated with a B-cell receptor inhibitor or if these medicines have stopped working, and for patients without these genetic alterations who have failed chemoimmunotherapy and a B‑cell receptor pathway inhibitor [9]. Second, venetoclax is approved in combination with the anti-CD20 antibody rituximab (VenR), as a fixed-duration regimen for pre-treated patients with CLL [10]. Third, venetoclax is approved in combination with obinutuzumab (VenO) or ibrutinib (VenI) in previously untreated patients with CLL [11, 12, 13].

Due to its potent apoptotic effect on lymphocytic cells, venetoclax treatment is associated with a risk for tumor lysis syndrome (TLS). TLS is a potentially serious oncologic emergency that can be triggered by spontaneous or therapy-induced rapid cell death [14, 15] The rapid release of cell contents can lead to hyperkalemia, hypocalcemia, hyperphosphatemia, and hyperuricemia, which may result in renal dysfunction, cardiac arrhythmias, and encephalopathy. In extreme cases, TLS can lead to seizures and death from multiorgan failure [16, 17]. TLS can be classified according to the Howard or Cairo-Bishop criteria as either laboratory or clinical TLS [18, 19]. Laboratory TLS according to the Howard criteria is present when two or more metabolic disturbances (hyperuricemia, hyperkalemia, hyperphosphatemia, or hypocalcemia) occur within a 24-hour period. Clinical TLS is present when, in addition to the laboratory TLS changes, other changes such as elevated creatinine, seizures, cardiac arrhythmias, or death occur [18].

In the first-in-human/phase 1b trials of venetoclax, TLS was reported as a serious side effect. In order to minimize the risk of TLS, a strict TLS risk stratification was developed along with dose ramp-up (20 mg, 50 mg, 100 mg, 200 mg,
400 mg) over 5 weeks (dose titration phase) [23]. Adherence to this dosing strategy, combined with TLS prophylaxis with hypouricemic drugs, hydration, and monitoring of TLS markers has allowed venetoclax to be safely administered in clinical trials [9, 10, 11, 12].

A comprehensive review of TLS incidence across venetoclax clinical trials demonstrated that patients with a high tumor burden (bulky lymph nodes ≥ 5 cm and/or elevated absolute lymphocyte count [ALC]) are at a higher risk when initiating venetoclax. Reduced renal function at screening (creatinine clearance < 80 mL/min) and concomitant medications such as CYP3A4 inhibitors further predispose patients to the risk of developing TLS [17, 20, 21, 22].

By following the recommended measures after initiating venetoclax, the incidence of laboratory-confirmed TLS within clinical trials in CLL was ∼1.1–3.8% with no cases of clinical manifestation [21, 24]. In contrast, retrospective real-world observations have reported higher rates of laboratory (5.7–6.3%) and clinical (2.7–6.3%) TLS events [25, 26, 27].

To determine the effectiveness and tolerability of venetoclax as monotherapy (Ven), or in combination with rituximab (VenR) or obinutuzumab (VenO) in patients with CLL under real-world conditions, we conducted a prospective non-interventional observational study (VeRVe) within a real-world clinical setting. These post-hoc analyses were conducted to determine the incidence and outcome of TLS events in these patients. The patient case reports discussed in the current analyses were documented during the study and clinical TLS events were reviewed and discussed in the context of patient-specific disease characteristics, venetoclax treatment initiation, and TLS risk-mitigation.

# Methods

## Study design and population

VeRVe is a prospective, international, multi-center observational study in patients with CLL who receive venetoclax as single agent or in combination with an anti-CD20 antibody. The study is being conducted across Austria, Germany, and Switzerland, and was designed to record and analyze real-world treatment data from daily clinical practice. All processes, treatments, procedures (ie, blood sampling), and diagnostic steps, including the times for follow-up examinations, were selected to correlate with daily routine practice. Informed consent was obtained from all participants included in the study.

## Procedures

Adult patients with CLL requiring therapy according to International Workshop on Chronic Lymphocytic Leukemia criteria and who initiated venetoclax therapy were eligible if treated as specified in the local label for any line of treatment. The regimen was prescribed at the discretion of the treating physician in accordance with local clinical practice. Treatment decisions were made independently and preceded the decision to offer the patient the opportunity to participate in this study.

All patient and disease characteristics were summarized descriptively at baseline prior to initiation of venetoclax therapy. Quantitative variables are presented as absolute number, mean, median, standard deviation, quartiles, minimum and maximum. Categorical/binary variables, such as sex, age groups, initial stage, etc., are presented by absolute numbers and percentages. TLS events were documented by the physician and recorded as laboratory and/or clinical TLS according to the definition by Howard et al. [18]. The study was conducted in accordance with local laws and regulations of the participating countries, and all patients signed informed consent.

## Statistical analyses

The study is ongoing at the time of this publication and the current analyses are based on a data cut-off from March 3, 2022. In these descriptive, retrospective analyses, adult patients with CLL requiring therapy who have received at least one dose of Ven, VenR, or VenO were included. Pearson’s method was used to test for a correlation between the occurrence of isolated metabolic abnormalities and TLS. Statistical analyses were performed using SAS version 9.4 (SAS Institute Inc., Cary, NC, USA) and R version 4.1.3 (R Foundation for Statistical Computing, Vienna, Austria) by the German Oncology group.

# Results

## Patient characteristics

A total of 239 patients who had received at least one dose of venetoclax were included in the current analyses. Seventy-eight (33%) patients were treated with Ven, 101 (42%) patients were treated with VenR and 60 (25%) received VenO. Median age at baseline was 74 and 73 years for the Ven and VenR groups, respectively, and 67 years in the VenO group. Patients treated with Ven or VenR received a median of 2 and 1 prior lines of treatment, respectively (range, 1–10). Prognostically unfavorable cytogenetics (ie, del(17p)/*TP53*^mut^) were more common in the group treated with Ven vs VenR or VenO (del[17p]: 44% vs. 28% or 24%; *TP53*^mut^: 49% vs. 30% or 15%, respectively) amongst those tested. Of those tested, the proportion of patients with unmutated IGHV was 73%, 69%, and 67% in the Ven, VenR, and VenO groups, respectively. Baseline characteristics prior to venetoclax treatment initiation are shown in Table 1.

Baseline characteristics of patients with (n = 28) or without (n = 200) occurrence of TLS are shown in Table 2. Generally, comorbidities were similar between both groups. Median leukocyte and absolute lymphocyte counts were higher in the TLS group compared with the non-TLS group (63 vs. 19.3×10^9^/L and 30.9 vs. 10.9×10^9^/L, respectively). Higher rates for both parameters were also observed among the 5 patients that developed clinical TLS. The median creatinine clearance was higher in the non-TLS group (Pearson’s method, *p* = 0.03). Data for lymph node size was not available for all patients, but the percentage of patients with lymph node size > 5cm was comparable in patients with or without TLS (17.9% vs. 18.5%, respectively). The percentage of patients who received allopurinol as a supportive agent at baseline was higher in the non-TLS compared with the TLS group (61.5% vs. 53.6%). Similarly, slightly more patients who experienced clinical TLS had received allopurinol at baseline compared with those patients with laboratory TLS (60.0% vs. 54.2%, respectively).

## Incidence of TLS

Twenty-eight (15.6%) cases of TLS were identified. Of these, 19 (10.6%) were documented as TLS in the electronic case report form (eCRF) and 9 (5%) were not documented as TLS but fulfilled the Howard criteria for TLS (Table 3). The incidence of laboratory TLS was higher in the VenR cohort compared with the Ven cohort (10.9% vs. 5%), whereas the rate of clinical TLS was lower in the VenR cohort compared with the Ven cohort (2% vs. 3.8%). As no cases of TLS were observed in the VenO arm and Obinutuzumab is dosed before Ven, resulting in debulking this subgroup of patients were excluded from the calculation of the incidence of TLS.

***Laboratory TLS***

Overall, the test results of 15 patients (8.4% of the eligible population) fulfilled the Howard criteria for laboratory TLS alone. One patient developed two laboratory TLS events; the first occurring 2 days after the first dose of 20 mg, and the second 32–33 hours after the first 200 mg dose.

***Clinical TLS***

Of the 19 TLS events which were documented in the eCRF, 5 met the criteria for clinical TLS. Three of these events occurred in patients treated with Ven and 2 cases in patients treated with VenR. Three patients required hospitalization during treatment (Ven:1; VenR:2). The median hospitalization time was 17 days. One patient already had an elevated uric acid level at therapy initiation. For the other 4 patients, blood chemistry was without remarkable findings at baseline. Table 4 gives an overview of the patient-specific characteristics for patients who experienced a clinical TLS.

TLS occurred in all patients during the early ramp-up phase, none of which was fatal or led to renal failure. No TLS event was documented as the reason for discontinuation of venetoclax. However, two patients with clinical TLS discontinued venetoclax treatment early due to other AEs (1 thrombocytopenia and 1 arrythmia). Three patients reached the final dose of 400 mg venetoclax, despite clinical TLS during the early ramp-up phase. For one patient with clinical TLS who reached the final dose of 400 mg, the ramp-up phase was shortened before and during occurrence of the TLS.

# Discussion

In the current analyses, 239 patients received at least one dose of venetoclax. Under real-world conditions, venetoclax given as monotherapy or in combination with rituximab is mainly prescribed to elderly pretreated patients. The median age of the patient population in the monotherapy and VenR combination arms was higher at baseline than in the pivotal monotherapy (M13-982, M14-032) [9, 28] and combination therapy trials (MURANO) [10] but younger in the VenO combination arm than the pivotal frontline therapy trial (CLL14) [29]. However, the median age at diagnosis (62 years) in this study was lower than the literature data published by Surveillance, Epidemiology, and End Results (SEER) Program (70 years) [30]. This indicates a longer duration of disease in our cohort. Adverse risk cytogenetics (del[17p]/*TP53*^mut^) were more common in the monotherapy group than in the combination groups, although they were generally comparable to previous real-world studies [26, 31].

The overall incidence of TLS in this study was 15.6%. In a large retrospective analysis, Seymour et al*.* [32] examined the rate of TLS cases in patients with CLL who received venetoclax-based regimens in frontline and relapsed/refractory diseases. Of 1,138 patients from eight clinical trials, 20 TLS (1.8%) events of any grade and 50 blood chemistry abnormalities, which met the Howard criteria of TLS, were reported. All TLS cases were transient, allowing affected patients to resume/continue therapy without permanent sequelae or death due to TLS. In contrast to data from clinical trials, the incidence rate of documented TLS cases (of any grade) within the real-world studies across the US and UK ranged from 4–15%. [25, 27, 33]. Clinical TLS rates ranged from 2– 6% [27, 33]. Across Europe, TLS was observed in 14 (22%) patients (clinical: 2 [3%]) receiving Ven in a French Innovative Leukemia Organization (FILO) study of the French compassionate use cohort [31]; in a Spanish retrospective observational trial (VENARES), 125 eligible patients treated with Ven (n = 71), VenR (n = 36), VenO (n = 5) or other Ven combination
(n = 13) were included in the analysis. TLS was reported in 4 (3.2%) patients during ramp up (3 laboratory, 1 clinical) with no patients discontinuing treatment due to TLS [34]. Thus, the overall documented TLS rate of 15.6% and clinical TLS rate of 2.8% (5/179 patients) found in this analysis is comparable to other retrospective real-world studies and prospective clinical trial data. In the study by Seymour et al. [32], authors analyzed additional data from the AbbVie Global Pharmacovigilance Database including post-marketing data from approximately 20,000 patients treated under real-world conditions. A total of 236 cases of TLS were reported including 17 cases with severe consequences (clinical sequelae) attributable to TLS. These patients had relapsed/refractory CLL and a median age of 69 years (range, 45–84). Multiple comorbidities were present, including renal insufficiency (n = 7), defined as creatinine clearance or glomerular filtration rate < 80 mL/min (at baseline) and/or reported history of renal impairment. Of the 17 severe cases (~0.1%), 13 were fatal and 4 required dialysis. Eight patients developed TLS at the first dose step of 20 mg. The TLS risk category at baseline was not reported for 7 patients. Despite having enough data on the 17 cases to conclude that the severe or fatal outcomes were attributable to TLS, some details regarding these cases were unavailable. The authors concluded that key factors contributing to TLS may have included non-adherence to label recommendations for TLS risk mitigation by the prescribers [32]. It is therefore important that prescribers follow recommended preventive measures, including risk assessment, guidelines on hydration and use of anti-hyperuricemic agents, blood chemistry monitoring with early intervention when needed, and dose modifications for drug-drug interactions.

In our study, no fatal TLS events were reported, and all cases were transient. Most patients resumed therapy without any irreversible sequelae. The majority of the clinical TLS events occurred at the early phase of dose escalation (20/50 mg). All patients had impaired renal function (GFR < 80 mL/min) at baseline which was also significantly lower in patients with compared to patients without a TLS event. Lymphocytes were increased at baseline, which is considered the most relevant predisposing factor for TLS. Although ALC and lymph node size are the only parameters currently deemed necessary to predict risk of TLS per label, previous analyses have demonstrated that baseline creatinine clearance can serve as a predictor of TLS [25]. In our study, no other baseline parameters were found to be predictive of the incidence of TLS. Additional measures of tumor bulk may also be important in assessing risk for TLS, including spleen size, extent of bone marrow involvement by CLL, serum lactate dehydrogenase, and β-2 microglobulin [27].

Based on available data and analyses, particularly from the real-world/post-marketing retrospective studies, the venetoclax summary of product characteristics (SmPC) was revised in June 2021 to reflect the updated recommendations and to emphasize the importance of strict adherence to the TLS mitigation measures in all CLL patients [13]. To minimize the risk, prescribing physicians should assess patient-specific factors for TLS risk, including comorbidities and particularly impaired renal function, tumor burden, and splenomegaly. Prophylactic fluid intake and the use of uric acid lowering medication (eg, allopurinol) is mandatory in all patients prior to the first dose of venetoclax. Laboratory blood chemistry values and tumor burden should be closely monitored, and the recommended dose adjustments and actions should be followed in the event of changes in blood counts or symptoms suggestive of TLS. These data allow for a richer understanding of the rates of clinical and laboratory TLS with venetoclax outside of the clinical trial setting.

In summary, multiple factors may give rise to the risk of TLS. Under real-world conditions in Austria, Germany, and Switzerland, venetoclax therapy initiation was well-tolerated and few clinical TLS events occurred, and none of them were fatal or lead to total renal failure. These findings highlight the importance of continuing to follow the current TLS mitigation protocol, ie, the five-week ramp-up, patient hydration, anti-hyperuricemic prophylaxis, and close monitoring of the patients’ blood chemistry in order to prevent the occurrence of a TLS event. Further research into biochemical factors that might be associated with TLS risk is warranted.

Future observations from this ongoing study will add to the body of evidence for use of TLS mitigation measures with venetoclax treatment as mono- and combination-therapy.

**Funding**

The study was funded by AbbVie Germany GmbH & Co KG.

**Author contribution** JH and IS designed the study; IS, CL, TW, BO, MZ, TN and DR collected the data; all authors interpreted the data. The first draft of the manuscript was written by Thomas Kuhn, and all authors commented on the first and subsequent versions of the manuscript. No honoraria or payments were made for authorship. AbbVie sponsored the study; contributed to the design; participated in the collection, analysis, and interpretation of data; in writing, reviewing, and approval of the final version. Material preparation, data collection, Statistical analyses were performed by Anna Eisen and Yves Djago from German Oncology GmbH with funding provided by AbbVie. All authors read and approved the final manuscript. Medical writing and editorial support were provided by Medical Expressions, London, with funding provided by AbbVie

AbbVie is committed to responsible data sharing regarding the clinical trials we sponsor. This includes access to anonymized, individual, and trial-level data (analysis data sets), as well as other information (eg, protocols, clinical study reports, or analysis plans), as long as the trials are not part of an ongoing or planned regulatory submission. This includes requests for clinical trial data for unlicensed products and indications.

These clinical trial data can be requested by any qualified researchers who engage in rigorous, independent, scientific research, and will be provided following review and approval of a research proposal, Statistical Analysis Plan (SAP), and execution of a Data Sharing Agreement (DSA). Data requests can be submitted at any time after approval in the US and Europe and after acceptance of this manuscript for publication. The data will be accessible for 12 months, with possible extensions considered. For more information on the process or to submit a request, visit the following link: https://www.abbvieclinicaltrials.com/hcp/data-sharing/Declarations

**Ethics approval** The study was approved by the ethics commission of Berlin

**Informed consent** Prior written consent was obtained for all patients enrolled in the study

**Competing interests** BO has received honoraria for participation in advisory boards from AbbVie. IS has received honoraria from AbbVie, Amgen, AstraZeneca, BeiGene, Janssen, Roche, Servier, CL received consulting fees from AbbVie, Amgen. TW received honoraria or research funding Novartis, Celgene, Roche, Bayer, BMS and AbbVie, DR has received honoraria or research grants from AbbVie, AstraZeneca, Gilead, Janssen, Verastem, Roche, Cellestia, TK, JH and KF are employees of AbbVie and may own AbbVie stock.

# Tables

## Table 1. Baseline Characteristics

|  | Total  (N=239) | Ven  (N=78) | VenR  (N=101) | VenO  (N=60) |
| --- | --- | --- | --- | --- |
| Sex, n |  |  |  |  |
| Male | 157 | 47 | 73 | 37 |
| Female | 82 | 31 | 28 | 23 |
| Age, years, median [range] | 73 [40–94] | 74 [40–94] | 73 [50–88] | 67 [43–87] |
| Binet stage^#^, n (%) |  |  |  |  |
| A | 54 (23) | 21 (27) | 21 (21) | 12 (20) |
| B | 92 (39) | 28 (36) | 37 (37) | 27 (45) |
| C | 92 (39) | 29 (37) | 43 (43) | 20 (33) |
| Missing | 1 | 0 | 0 | 1 |
| Del(17p), n (%)* |  |  |  |  |
| Deleted | 63 (26) | 30 (38) | 23 (23) | 10 (17) |
| Not deleted | 129 (54) | 38 (49) | 59 (58) | 32 (53) |
| Missing | 47 (20) | 10 (13) | 19 (19) | 18 (30) |
| *TP53*, n (%)* |  |  |  |  |
| Mutated | 62 (26) | 31 (40) | 24 (24) | 7 (12) |
| Unmutated | 127 (53) | 32 (41) | 56(55) | 39 (65) |
| Missing | 50 (21) | 15 (19) | 21 (21) | 14 (23) |
| IGHV status, n (%)* |  |  |  |  |
| Mutated | 41 (17) | 10 (13) | 18 (18) | 13 (22) |
| Unmutated | 93 (39) | 27 (35) | 40 (40) | 26 (43) |
| Missing | 105 (44) | 41 (53) | 43 (43) | 21 (35) |
| Prior therapies, n | 179 | 78 | 101 | 0 |
| Median [range] | 2 [1–10] | 2 [1–10] | 1 [1–10] | 0 |
| Patients with comorbidities, n (%) | 177 (74) | 62 (26) | 77 (32) | 38 (16) |

*Percentage of patients tested.

^#^Binet staging system for CLL: Stage A, fewer than 3 groups of swollen (enlarged) lymph nodes. Stage B, 3 or more groups of enlarged lymph nodes. Stage C, low number of red blood cells or platelets.

Del, deletion; IGHV, immunoglobulin heavy chain variable region gene; Ven, venetoclax monotherapy; VenR, venetoclax and rituximab; VenO, venetoclax and obinutuzumab.

##

## Table 2. Summary of baseline parameters between TLS and non-TLS patients

| **Parameter** | **All patients** | **Patients  without TLS*** | **Patients with TLS*** | **Patients with laboratory TLS** | **Patients with clinical TLS** |
| --- | --- | --- | --- | --- | --- |
| Patients, n/N | 239 | 200 | 28 | 24 | 5 |
| Age, years, median [range] | 73 [40–94] | 73 [40–94] | 75 [54–88] | 74.5 [54–84] | 76 [54–88] |
| Leukocytes, ×10^9^/L, median [range] | N=229  19.3 [1.4–407] | N=194  19.3 [1.4–407] | N=26  63 [6.4–254.8] | N=22  46 [6.4–254.8] | N=5  121 [44–242] |
| Lymphocytes, ×10^9^/L, median [range] | N=175  10.9 [0.2–287.1] | N=149  10.9 [0.2–287.1] | N=19  30.9 [0.8–125] | N=16  30.2 [0.8–125] | N=3  69.9 [0.8–94] |
| Creatinine, mg/dL, median [range] | N=229  1.02 [0.42–3.06] | N=194  1.01 [0.42–3.06] | N=26  1.2 [0.7–2.3] | N=22  1.3 [0.7–2.3] | N=5  1.2 [1.0–1.4] |
| Creatinine clearance, mL/min, median, [range] | N=225  68.2 [20.3–214.6] | N=192  69.3 [20.3–214.6] | N=26  56.7 [25.9–104.4] | N=22  59.4 [25.9–104.4] | N=5  59 [33.2–76.9] |
| Potassium, mmol/L, median [range] | N=222  4.3 [2.26–5.9] | N=188  4.3 [2.26–5.7] | N=25  4.4 [3.4–5.9] | N=21  4.4 [3.4–5.9] | N=5  4.3 [3.8–4.9] |
| Calcium, mmol/L, median [range] | N=210  2.3 [0.53–4.8] | N=177  2.3 [0.53–4.8] | N=25  2.3 [2.01–2.6] | N=21  2.3 [2.02–2.6] | N=5  2.3 [2.0–2.5] |
| Phosphate, mmol/L, median [range] | N=137  1.08 [0.5–1.95] | N=114  1.08 [0.58–1.95] | N=18  1.1 [0.5–1.4] | N=15  1.1 [0.5–1.4] | N=4  1.2 [0.8–1.4] |
| Del(17p)/*TP53*^mut^, n/N (%) | 85/215 (39.5) | 72/179 (40) | 11/27 (41) | 11/23 (47.8) | 0/5 (0) |
| IGHV^unmut^, n/N (%) | 93/134 (69.4) | 79/200 (39.5) | 12/12 (100) | 11/11 (100) | 1/1 (100) |
| Lymph node size |  |  |  |  |  |
| Patients assessed, n (%) | 94 (39.3) | 82 (41.0) | 10 (35.7) | 8 (33.3) | 2 (40.0) |
| Size > 5 cm, n (%) | 42 (17.6) | 37 (18.5) | 5 (17.9) | 5 (20.8) | 0 |
| Comorbidities, n |  |  |  |  |  |
| Any | 182 | 148 | 22 | 19 | 4 |
| Renal | 28 | 21 | 5 | 4 | 1 |
| Cardiovascular | 128 | 108 | 15 | 15 | 0 |
| Metabolic/Endocrine | 74 | 60 | 10 | 9 | 1 |
| Supportive medication, n |  |  |  |  |  |
| Any | 172 | 129 | 15 | 14 | 1 |
| TLS prophylaxis, n |  |  |  |  |  |
| Allopurinol | 162 | 123 | 15 | 13 | 3 |
| Rasburicase | 10 | 8 | 2 | 1 | 0 |
| Febuxostat | 5 | 4 | 1 | 1 | 1 |

*Patients who had documented laboratory parameters at baseline, no data available for 11 patients.

Del, deletion; IGHV, immunoglobulin heavy chain variable region gene; TLS, tumor lysis syndrome.

| Event | Total | Ven | VenR |  |
| --- | --- | --- | --- | --- |
| TLS events, n (%) | 28 (11.7)^#^ | 12 (15.3) | 16 (15.8) |  |
| Laboratory TLS*^,#^ | 24 (10)^#^ | 9 (11.5) | 15 (14.9) |  |
| Clinical TLS | 5 (2.1) | 3 (3.8) | 2 (2) |  |
| With treatment | 10 (52.6) | 4 (57) | 6 (50) |  |
| With hospitalization | 6 (31.6) | 1 (14.3) | 5 (41.7) |  |
| Event | Total | Ven | VenR | |
| TLS events, n (%) | 28 (15.6)^#^ | 12 (15.3) | 16 (15.8) | |
| Laboratory TLS*^,#^ | 15 (8.4)^#^ | 4 (5) | 11 (10.9) | |
|  |  |  |  | |
| Clinical TLS | 5 (2.8) | 3 (3.8) | 2 (2) | |
| With treatment | 10 (52.6) | 4 (57) | 6 (50) | |
| With hospitalization | 6 (31.6) | 1 (14.3) | 5 (41.7) | |

## Table Table 3. Incidence of TLS

*Cases with blood chemistry values that fulfilled the Howard criteria.

^#^Including one patient with two laboratory TLS events.

TLS, tumor lysis syndrome; Ven, venetoclax monotherapy; VenR, venetoclax and rituximab; VenO, venetoclax and obinutuzumab.

## Table 4. Patients with clinical TLS

| **Patient ID** | **Dose at which TLS occurred, mg** | **Regimen** | **Hospitalized** | **Lymphocytes, cells/µL*** | **Leukocytes, cells/µL*** | **Creatinine clearance, mL/min*** | **Age, years** | **Short ramp-up** | **Discontinued** |
| --- | --- | --- | --- | --- | --- | --- | --- | --- | --- |
| 1 | 100 | Ven | No | 94,000 | 242.0 | 34.4 | 80 | Yes | No |
| 2 | 50 | VenR | Yes, day 24 | N/A | 120.6 | 65.8 | 54 | Yes | No |
| 3 | 20 | Ven | Yes, day 17 | 840 | 44.3 | 60.8 | 76 | No | Yes |
| 4 | 20 | VenR | Yes, day 8 | N/A | 139.0 | 45.3 | 88 | No | Yes |
| 5 | 20 | Ven | No | 69,900 | 78.9 | 79.2 | 71 | No | No |

*At baseline. Normal range for lymphocytes is 1,500–3,000 cells/µL and leukocytes is 4,000–10,000 cells/µL. For creatinine, a value between 1–1.5 mL/min corresponds to normal renal function.

TLS, tumor lysis syndrome; Ven, venetoclax monotherapy; VenR, venetoclax and rituximab.

**References**

1. Punnoose EA, Leverson JD, Peale F et al (2016) Expression Profile of BCL-2, BCL-XL, and MCL-1 Predicts Pharmacological Response to the BCL-2 Selective Antagonist Venetoclax in Multiple Myeloma Models. Mol Cancer Ther 15:1132–1144. https://doi.org/10.1158/1535-7163.MCT-15-0730

2. Leverson JD, Sampath D, Souers AJ et al (2017) Found in translation: How preclinical research is guiding the clinical development of the BCL2-selective inhibitor venetoclax. Cancer Discov 7:1376–1393. https://doi.org/10.1158/2159-8290.CD-17-0797

3. Billard C (2014) Apoptosis inducers in chronic lymphocytic leukemia. Oncotarget 5:309–325. https://doi.org/10.18632/oncotarget.1480

4. Cory S, Huang DC, Adams JM (2003) The Bcl-2 family: roles in cell survival and oncogenesis. Oncogene 22:8590–8607. https://doi.org/10.1038/sj.onc.1207102

5. Deng J, Carlson N, Takeyama K et al (2007) BH3 profiling identifies three distinct classes of apoptotic blocks to predict response to ABT-737 and conventional chemotherapeutic agents. Cancer Cell 12:171–185. https://doi.org/10.1016/j.ccr.2007.07.001

6. Plati J, Bucur O, Khosravi-Far R (2011) Apoptotic cell signaling in cancer progression and therapy. Integr Biol (Camb) 3:279–296. https://doi.org/10.1039/c0ib00144a

7. Souers AJ, Leverson JD, Boghaert ER et al (2013) ABT-199, a potent and selective BCL-2 inhibitor, achieves antitumor activity while sparing platelets. Nat Med 19:202–208. https://doi.org/10.1038/nm.3048

8. Klanova M, Klener P (2020) BCL-2 Proteins in Pathogenesis and Therapy of B-Cell Non-Hodgkin Lymphomas. Cancers (Basel) 12(4), 938. https://doi.org/10.3390/cancers12040938

9. Stilgenbauer S, Eichhorst B, Schetelig J et al (2016) Venetoclax in relapsed or refractory chronic lymphocytic leukaemia with 17p deletion: a multicentre, open-label, phase 2 study. Lancet Oncol 17:768–778. https://doi.org/10.1016/S1470-2045(16)30019-5

10. Seymour JF, Kipps TJ, Eichhorst B et al (2018) Venetoclax-rituximab in relapsed or refractory chronic lymphocytic leukemia. N Engl J Med 378:1107–1120. https://doi.org/10.1056/NEJMoa1713976

11. Fischer K, Al-Sawaf O, Bahlo J et al (2019) Venetoclax and obinutuzumab in patients with CLL and coexisting conditions. N Engl J Med 380:2225–2236. https://doi.org/10.1056/NEJMoa1815281

12. Venclyxto^®^ (venetoclax) (2022) Summary of Product Characteristics. European Medicines Agency. <https://www.ema.europa.eu/en/documents/product-information/venclyxto-epar-product-information_en.pdf> Accessed 09 May 2023

13. Venclyxto. European Medicines Agency. <https://www.ema.europa.eu/en/medicines/human/EPAR/venclyxto>. Accessed 09 May 2023

14. Hochberg J, Cairo MS (2008) Tumor lysis syndrome: current perspective. Haematologica 93:9–13. https://doi.org/10.3324/haematol.12327

15. Tosi P, Barosi G, Lazzaro C et al (2008) Consensus conference on the management of tumor lysis syndrome. Haematologica 93:1877–1885.

16. Howard SC (20140Tumor lysis syndrome. In: Abeloff's Clinical Oncology, 5th edn. Churchill Livingstone pp591–596. <https://doi.org/10.1016/B978-1-4557-2865-7.00038-2>

17. Cheson BD, Heitner Enschede S, Cerri E et al (2017) Tumor Lysis Syndrome in Chronic Lymphocytic Leukemia with Novel Targeted Agents. Oncologist 22:1283–1291. https://doi.org/10.1634/theoncologist.2017-0055

18. Howard SC, Jones DP, Pui CH. The tumor lysis syndrome. N Engl J Med 2011;364:1844–54. https://doi.org/10.3324/haematol.13290

19. Cairo MS, Bishop M (2004) Tumour lysis syndrome: new therapeutic strategies and classification. Br J Haematol 127:3–11. https://doi.org/10.1111/j.1365-2141.2004.05094.x

20. Gribben JG (2020) Practical management of tumour lysis syndrome in venetoclax-treated patients with chronic lymphocytic leukaemia. Br J Haematol 188:844–851. https://doi.org/10.1111/bjh.16345

21. Fischer K, Al-Sawaf O, Hallek M (2020) Preventing and monitoring for tumor lysis syndrome and other toxicities of venetoclax during treatment of chronic lymphocytic leukemia. Hematology Am Soc Hematol Educ Program 2020(1):357–362. https://doi.org/10.1182/hematology.2020000120

22. Jones GL, Will A, Jackson GH et al (2015) British Committee for Standards in Haematology Guidelines for the management of tumour lysis syndrome in adults and children with haematological malignancies on behalf of the British Committee for Standards in Haematology. Br J Haematol 169:661–671. https://doi.org/10.1111/bjh.13403

23. Roberts AW, Davids MS, Pagel JM et al (2016) Targeting BCL2 with Venetoclax in Relapsed Chronic Lymphocytic Leukemia. N Engl J Med 374:311–322. https://doi.org/10.1056/NEJMoa1513257

24. Tambaro FP, Wierda WG (2020) Tumour lysis syndrome in patients with chronic lymphocytic leukaemia treated with BCL-2 inhibitors: Risk factors, prophylaxis, and treatment recommendations. Lancet Haematol 7:e168–e176. https://doi.org/10.1016/S2352-3026(19)30253-4

25. Roeker LE, Fox CP, Eyre TA et al (2019) Tumor Lysis, Adverse Events, and Dose Adjustments in 297 Venetoclax-Treated CLL Patients in Routine Clinical Practice. Clin Cancer Res 25:4264–4270. https://doi.org/10.1158/1078-0432.CCR-19-0361

26. Mato AR, Thompson M, Allan JN et al (2018) Real-world outcomes and management strategies for venetoclax-treated chronic lymphocytic leukemia patients in the United States. Haematologica 103:1511–1517. https://doi.org/10.3324/haematol.2018.193615

27. Koehler AB, Leung N, Call TG et al (2020) Incidence and risk of tumor lysis syndrome in patients with relapsed chronic lymphocytic leukemia (CLL) treated with venetoclax in routine clinical practice. Leuk Lymphoma 61:2383–2388. https://doi.org/10.1080/10428194.2020.1768384

28. Jones JA, Mato AR, Wierda WG et al (2018) Venetoclax for chronic lymphocytic leukaemia progressing after ibrutinib: an interim analysis of a multicentre, open-label, phase 2 trial. Lancet Oncol 19:65–75. https://doi.org/10.1016/S1470-2045(17)30909-9

29. Al-Sawaf O, Zhang C, Tandon M et al (2020) Venetoclax plus obinutuzumab versus chlorambucil plus obinutuzumab for previously untreated chronic lymphocytic leukaemia (CLL14): Follow-up results from a multicentre, open-label, randomised, phase 3 trial. Lancet Oncol 21:1188–1200. https://doi.org/10.1016/S1470-2045(20)30443-5

30. Surveillance, Epidemiology, and End Results (SEER) Program, Cancer Stat Facts: Chronic Lymphocytic Leukemia. National Cancer Institute. <https://seer.cancer.gov/statfacts/html/clyl.html>. Accessed 09 May 2023

31. Bouclet F, Calleja A, Dilhuydy MS et al (2021) Real-world outcomes following venetoclax therapy in patients with chronic lymphocytic leukemia or Richter syndrome: a FILO study of the French compassionate use cohort. Ann Hematol 100:987–993. https://doi.org/10.1007/s00277-021-04419-w

32. Seymour JF, Gribben JG, Davids MS et al (2020) Assessment of tumor lysis syndrome in patients with chronic lymphocytic leukemia treated with venetoclax in the clinical trial and post-marketing settings. Blood 136 (Suppl 1):37–38. https://doi.org/10.1182/blood-2020-134938

33. Mora RF, Rampotas A, Halperin D et al (2023)Venetoclax ramp-up strategies for chronic lymphocytic leukaemia in the United Kingdom: a real world multicentre retrospective study. Br J Haematol. https://doi: 10.1111/bjh.18738.

34. Ferra C, Terol MJ, Palomanes JM et al (2022) Efficacy and safety of treatment venetoclax monotherapy or combined with rituximab in patients with relapsed/refractory chronic lymphocytic leukemia (CLL) in the real world setting in Spain; An update of the Venares study. Blood 140 (Suppl 1):9908–9910. https://doi.org/10.1182/blood-2022-162186
